# Application of the Deep Inspiration Breath-Hold Technique in Proton Therapy for Mediastinal Lymphomas: Initial Experience

**DOI:** 10.3390/cancers17121985

**Published:** 2025-06-14

**Authors:** Magdalena Garbacz, Tomasz Skóra, Anna Cepiga, Gabriela Foltyńska, Jan Gajewski, Eleonora Góra, Dominika Kędzierska-Pardel, Wiktor Komenda, Dawid Krzempek, Emilia Krzywonos, Tomasz Mikołajski, Antoni Ruciński, Karolina Sobkowicz, Urszula Sowa, Agnieszka Wochnik, Kamil Kisielewicz, Renata Kopeć

**Affiliations:** 1 Cyclotron Centre Bronowice, The Henryk Niewodniczański Institute of Nuclear Physics Polish Academy of Sciences, ul. Radzikowskiego 152, 31-342 Krakow, Poland; 2 Maria Sklodowska-Curie National Research Institute of Oncology, Krakow Branch, ul. Garncarska 11, 31-115 Krakow, Poland; 3 Faculty of Medical Sciences in Zabrze, Medical University of Silesia, ul Poniatowskiego 15, 40-055 Katowice, Poland

**Keywords:** deep inspiration breath hold, mediastinal lymphoma, surface-guided radiotherapy in proton therapy

## Abstract

**Background:** This work presents the procedures and application of the deep inspiration breath-hold (DIBH) technique for mediastinal lymphoma patients at a proton therapy (PT) center. It also discusses the implementation and validation of the surface-guided radiotherapy (SGRT) protocol in terms of positioning accuracy. **Methods:** This study included six lymphoma patients. Dedicated computed tomography (CT) protocols and a treatment workflow based on international guidelines were developed. Clinical data from the treatment planning system (TPS) were used to assess the difference between DIBH and free-breathing irradiation. Additionally, data from an optical patient positioning system and kilovoltage (kV) imaging system were used to estimate positioning shifts. The new CT protocol reduced the volume CT dose index by over six times compared with the standard protocol. **Results**: The DIBH method decreased the mean dose to the heart and lungs by up to 7.02 Gy(RBE) and 0.83 Gy(RBE), respectively. The median magnitude of patient setup errors and repeatability in DIBH positioning was 0.4 cm and 0.18 cm (mean for males and females) for the SGRT protocol. The kV imaging showed a setup error of over 0.3 cm for both groups. **Conclusions**: Despite the small size of the patient cohort, the relatively large number of individual positioning sessions enabled the detection of statistically significant differences (*p* < 0.05) in certain areas between male and female patients; however, no significant difference in the displacement vector magnitude was observed. DIBH treatment with SGRT offers high reproducibility for patient positioning.

## Simple Summary

The aim of this work is to present the use of the deep inspiration breath-hold technique for mediastinal lymphoma patients treated with proton therapy. This study also explores the use of a patient positioning system to improve treatment accuracy. Six patients participated in this study. Special imaging protocols and treatment workflows were developed, and data from the treatment planning system and other tools helped assess the differences between the deep inspiration breath-hold technique and traditional treatment. The new imaging protocol reduced radiation doses compared with the standard protocol, and the deep inspiration breath-hold technique lowered the dose delivered to the heart and lungs. Additionally, the patient positioning system showed high reproducibility, with differences observed between men and women.

## 1. Introduction

### 1.1. Proton Therapy for Mediastinal Tumors

Pencil beam scanning (PBS) is a modern and highly precise proton therapy technique that enables optimal sparing of organs at risk (OAR). Its ability to conform the dose distribution to complex tumor geometries makes it especially suitable for cases where the target volume is located near critical structures. PBS is widely used in the treatment of tumors in anatomically sensitive areas such as the central nervous system, including the brain and adjacent regions, as well as in mediastinal tumors [1]. Several studies have demonstrated the benefits of using proton beam therapy for the treatment of mediastinal tumors, compared with conventional photon therapy [2,3,4]. However, irradiating moving tumors in the mediastinum with PBS often requires the use of an inclined board to improve chest visibility, as well as systems that track chest motion and, indirectly, tumor movement. Safe and effective irradiation of patients with mediastinal lymphoma can be ensured by assessing the mobility of the irradiated area and using adequate safety margins for the treated volume. Due to the shallow location and movement of the mediastinal tumors, a range shifter (RS) is typically used for irradiation, as well as increased layer spacing and volumetric repainting. There are three primary methods of irradiating patients with mediastinal tumors, depending on tumor mobility and patient capabilities: (1) free breathing (FB), (2) respiratory gating, and (3) breath hold (BH) [5,6]. Observations of the respiratory cycle during beam delivery can be addressed using optical systems or spirometric methods [7]. Each approach presents distinct benefits and limitations. During FB, the patient does not become tired during irradiation, and it requires delivering the treatment dose over a larger volume to cover the entire tumor during each respiratory phase. Gating also requires minimal effort from the patient, but irradiation can take a significantly longer time, since the irradiation occurs only during the selected breathing phase. Deep inspiration breath hold (DIBH) can reduce the dose to the organ at risk (OAR) and achieve nearly static dose delivery, but not all patients are able to maintain breath-hold for about 30 s to allow proper CT acquisition and irradiation.

### 1.2. Epidemiological Background

Currently, the incidence of Hodgkin and non-Hodgkin lymphomas in Poland is around 21 per 100,000 inhabitants, including women and men, and a large proportion of these are adolescents and young adults. Hodgkin lymphoma (HL) accounts for over 18% of all lymphoma cancer cases (about 8% of all diagnosed lymphoid, hematopoietic and related tissue) in Poland. In 2020, Hodgkin and non-Hodgkin lymphoma were diagnosed in 3709 people and resulted in 1857 deaths, across both genders, according to the National Cancer Registry [8]. Classical Hodgkin lymphoma has two peaks of incidence—the first peak usually occurs between ages 25 and 30, and the second peak is usually observed between ages 50 and 55. Non-classical Hodgkin lymphoma also has two peaks of incidence—the first typically occurs in childhood and the second between the ages of 30 and 40. In both cases, male morbidity is higher than female, with a 1.3:1 (male–female) ratio in classical Hodgkin lymphoma and approximately 3:1 in all cases of non-classical Hodgkin lymphoma [9]. Different therapeutic protocols are used depending on the stage of the disease and the presence of risk factors and treatment center preference, but chemotherapy combined with consolidation radiotherapy is generally the standard approach. ABVD chemotherapy (adriamycin + bleomycin + vinblastine + dacarbazine) is a cyclic protocol usually used for classical Hodgkin lymphoma. If risk factors are present, it is highly recommended to combine the ABVD protocol with the BEACOPP (bleomycin + etoposide + doxorubicin + cyclophosphamide + vincristine + procarbazine + prednisolone) protocol. This combination of chemotherapy protocols is also used in patients with advanced stages of the disease. Primary mediastinal B-cell lymphoma (PMBCL) accounts for about 2–3% of all diagnosed non-Hodgkin’s lymphomas and about 6–10% of all malignant B-cell lymphomas. This type of lymphoma generally occurs in young patients between 30 and 40 years old. The incidence rate is higher within females, with a ratio of 2:1 up to 3:1 in comparison with males [10]. Currently there is no specified, optimal treatment protocol. Application of the R-CHOP protocol improves complete remission rates in comparison to the CHOP protocol (84% for R-CHOP, 54% for CHOP, *p* = 0.015). R-CHOP also improves the event-free survival rate compared with the CHOP protocol (78% versus 54%, *p* = 0.012) [10]. The radiotherapy targets (post chemotherapy) depend on the lymph node groups involved before chemotherapy and on the response to prior treatment. Prescribed doses (20–36 Gy) are delivered in conventional fractionation schemes (2 Gy per fraction) in both classical and non-classical forms of Hodgkin lymphoma. In the recurrent form of HL, radiotherapy is administered at the same doses as in the classical and non-classical form, but only to previously unirradiated areas of the lymph nodes [9]. For PMBCL the total doses from radiation therapy can be up to 50 Gy in case of consolidation after chemoimmunotherapy or between 40 and 55 Gy for refractory disease, according to NCCN guidelines [11]. The recent ESTRO-ACROP guidelines [12] on the implementation of BH techniques stress the importance and complexity of this method for treating thoracic patients. This paper provides extensive data on the implementation of DIBH at a proton therapy facility, offering valuable information regarding the benefits of this method and the implemented workflow, which may be of interest to other facilities. This study focuses on presenting dosimetric differences of using the DIBH technique versus FB irradiation, and compares positioning accuracy using kV imaging and an optical system. 

## 2. Materials and Methods

### 2.1. Patients Data

Data from six patients with mediastinal lymphomas treated with the DIBH technique in 2023 were analyzed. Treatment consisted of chemotherapy followed by proton radiotherapy. Patient demographics and treatment details are summarized in Table 1.

### 2.2. Equipment for Treating Moving Targets/Mediastinal Tumors at CCB

**Figure 1:**
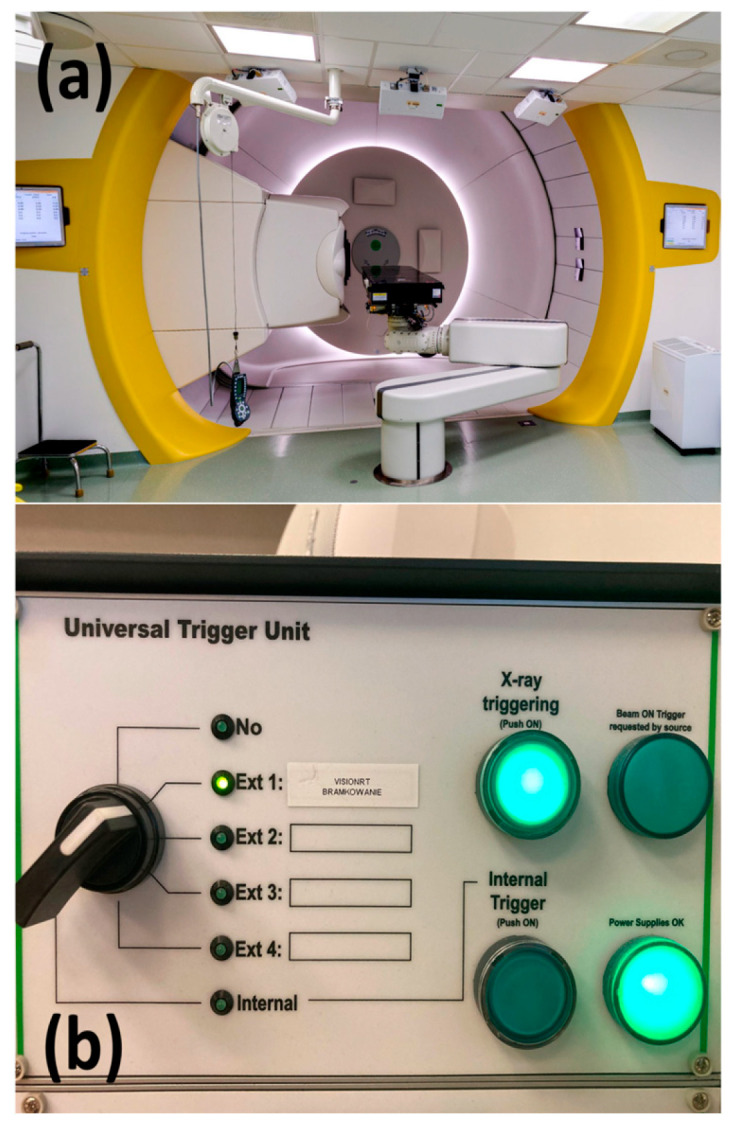
(**a**) The gantry room with VisionRT cameras. (**b**) Universal trigger unit panel enabling beam gating.

The Cyclotron Centre Bronowice (CCB) in Krakow is equipped with an IBA (Ion Beam Application, Louvain-La-Neuve, Belgium) cyclotron, which provides a proton scanning beam with a maximum energy of 226.1 MeV. Additionally, for surface-guided radiation therapy (SGRT), an AlignRT system (VisionRT Ltd., London, UK) is used. The system uses visible red light projected on the patient to acquire a three-dimensional image of the patient’s surface. This system notifies the user of any positional changes with an accuracy of 1 mm [13]. It is capable of being used in breath-hold (BH) irradiation. In 2023, CCB treated 24 mediastinal lymphoma patients, accounting for nearly 8% of all treated patients. Mediastinal lymphoma patients scheduled for proton therapy at CCB are qualified for deep inspiration breath-hold (DIBH) treatment at the Maria Skłodowska-Curie National Research Institute of Oncology in Krakow. Proton therapy using the DIBH technique is implemented at CCB with AlignRT real-time 3D surface imaging for patient positioning and verification. The VisionRT system, installed in each gantry room (see Figure 1a), is fully integrated with the IBA therapy control system via the universal beam triggering interface (UBTI), accessible through the universal trigger unit (UTU) in each control room (see Figure 1b). A reference chest wall surface is recorded during patient positioning using kilovoltage (kV) imaging in DIBH. A KUKA (Augsburg, Germany) robotic arm is used for patient positioning with six degrees of freedom (6DoF).

### 2.3. Patient and Treatment Plan Preparation

#### 2.3.1. Immobilization

**Figure 2:**
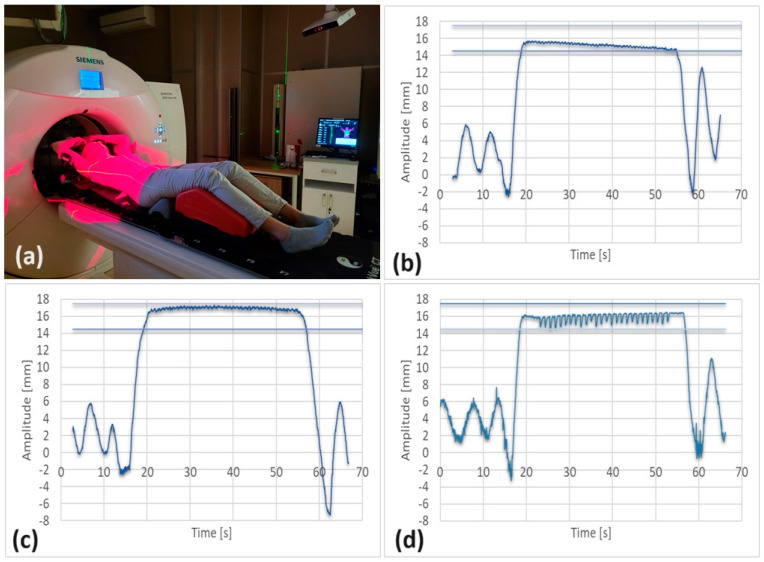
(**a**) Fixed patient position, (**b**,**c**) breath-hold curves from training, (**d**) breath-hold curve during CT (showing influence of CT table movements).

Patients undergoing radiotherapy with the DIBH technique were immobilized using a breast board ( Access™ Supine Breast & Lung, Qfix^®,^ Avondale, PA, USA) attached to a standard flat treatment table with indexing bars. The breast board surface was inclined at 10° to 15°, depending on the skin surface visibility in the irradiation area, as detected by the VisionRT cameras. This visibility was verified during setup and the breast board tilt was adjusted if needed. The patient’s arms were positioned overhead and immobilized with adjustable supports to ensure optimal therapeutic positioning (see Figure 2a). A bottom stopper on the breast board prevented the patient from sliding.

For neck lymph node irradiation, short thermoplastic masks were used with headrests, attached to the breast board via an adapter. For irradiating the supraclavicular region or areas below it, thermoplastic masks were omitted for patient comfort.

#### 2.3.2. Breath-Hold Training and CT with Surface Imaging

Patients underwent breathing training supported by AlignRT 6.3 software. Radiation therapists guided the training, acquired CT images, and prepared the patients for irradiation. During training, patients are asked to hold their breath for 30 s. If the minimum breath-hold (BH) time was not achieved or the patient struggled to maintain a stable BH, FB treatment was offered. Before treatment planning, two DIBH CT scans and one FB CT scan were acquired. Breathing training lasted 3–5 days, during which the breath-hold amplitude was verified as remaining within tolerance (±3 mm). Movement across other axes was monitored according to shifts and rotation margins. The patient’s ability to return to the pre-inhalation position was also assessed. On the last training day, low-dose CT scans (70 kV) were performed to verify breath-hold reproducibility and patient anatomy. Full-dose CT scans were used for treatment planning. The average dose reduction in CT was 6.66 times (CTDIvol) using the low-dose protocol. Protocol parameters are shown in Appendix A (Table A1).

Figure 2a shows a patient in a fixed position in the CT room, with the cameras activated to register the reference surface before applying the BH technique. Figure 2b,c present the BH curves obtained during the patient’s positioning and training. Figure 2d displays a characteristic BH waveform associated with the movements of the CT table.

Once the patient had been trained, full-dose FB and DIBH planning CT scans were acquired. During treatment, 1–2 low-dose CT scans were applied to monitor anatomical changes and ensure the treatment plan’s stability.

#### 2.3.3. Contouring

The reference CT image for treatment planning was the DIBH full-dose scan. The radiotherapy physician delineated the target structures (GTV, CTV, PTV) and organs at risk (OARs). CTV contours were also delineated on DIBH low-dose scans to assess target movement, which informed the treatment planning. The sum of CTVs comprised the internal target volume (ITV). The PTV was created by adding appropriate margins to the ITV or CTV based on internal motion and beam range uncertainty. Suggested margins for CTV-to-ITV or CTV-to-PTV range from 8–15 mm [14,15,16,17]. For DIBH, an 8 mm ITV-PTV margin was used, with an additional isotropic margin of 3 mm for range uncertainties. For FB plans, a 15 mm setup margin in the superior–inferior direction and 10 mm in other directions was used, plus an additional 3 mm margin for range uncertainties.

#### 2.3.4. Treatment Planning

Treatment planning was performed using Eclipse 16.1 (Varian Medical Systems, Palo Alto, CA, USA). To mitigate the interplay effect, a range shifter (RS) is often used to widen the pencil beam and improve plan robustness [18,19]. For mediastinal treatment, 3–4 treatment fields with gantry angles between 330° and 30° were typically used. In female patients, care was taken to avoid breast irradiation. Additional beams and/or isocenters were used when required. PTV coverage was ensured by adding technical PTV margins and performing robustness evaluations using ancillary dose calculations with perturbations. Robustness evaluation in general was based on ±3.5% uncertainty for CT calibration and a ±5 mm displacement in each direction. Target coverage of D90% ≥ 95% for ITV was the main robustness criterion [4].

#### 2.3.5. Proton Irradiation with Breath Holding

Patient positioning began by aligning the patient to the DICOM surface from the FB scan using the optical system. The next steps of the procedure were conducted during patient’s DIBH. Digitally reconstructed radiographs (DRRs) from CT DIBH full-dose images were uploaded, and the patient was aligned using both the optical system and X-ray flat panels. For positioning, orthogonal setup fields (anterior–posterior (AP) and lateral) were used. Due to the patient’s raised hands, the visibility of the anatomy in these fields was sufficient to assess the depth of inspiration by evaluating the position of the vertebral bodies and the sternum. For hands positioned along the body, a pair of orthogonal X-rays but from oblique (45°, 315°) should be considered. In addition, the position of the clavicles on the AP images was corrected in the case of PTV involving cervical lymph nodes. Irradiation started once the patient’s position matched the planning CT images (typically: ±2 mm for translations and ±1° rotation, as verified by kV imaging). Additionally, optical monitoring was performed continuously during treatment delivery to ensure that the radiation beam was activated only when the patient remained within the predefined positional tolerance.

### 2.4. Study I Design—DIBH vs. FB Dose Comparison

Each of the six patients included in the analysis was treated using DIBH technique at the CCB. For the purpose of this study, additional FB treatment plans were prepared for comparative analysis. The structures for FB treatment planning were contoured by the physicians as described in Section 2.3.3. To compare the treatment plans, DVH (dose-volume histogram) indices were collected, including D98 for CTV/ITV/PTV coverage, D2 for PTV, and the mean dose for selected OARs. Clinical goals for OARs were based on the NCCN guidelines [11]. Both DIBH (clinical) and FB (for research purposes) plans were designed to meet the same ITV and CTV target coverage and OAR dose constraints.

#### Statistical Analysis

The data were analyzed for statistical significance. The Shapiro–Wilk test [20] was used to assess normality. Differences between groups were evaluated using a *t*-test for normally distributed data [21] and the Wilcoxon test for non-normal distributions [22]. *p*-values were calculated using the SciPy 1.15.2 library in Python 3.10.12 [23], with *p* < 0.05 considered statistically significant.

### 2.5. Study II Design—Breath-Hold Duration and Patient Position Variability

**Figure 3:**
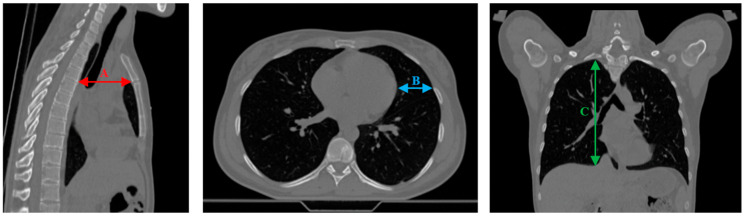
Measured anatomical distances (A, B, C—see text) used for breath-hold assessment.

CT scans provide baseline information on the accuracy of reproducing the therapeutic position during subsequent setups. Both full-dose and low-dose scans were used to compare CT-derived distances, similar to measurements performed in a previous study [24]. Measurements were made by a single physician to minimize interobserver variability. The following distances were compared (based on TPS contours): A. sternum to thoracic vertebral surface, B. rib to heart (mid-height), C. apex of lung to diaphragm (see Figure 3). These measurements were performed on all available DIBH CT images (both full and low-dose scans).

The reports from the optical system and patient treatment records were used to investigate patient position variability. Translations and rotations were obtained using AlignRT treatment session reports (SGRT FB setup error, SGRT DIBH setup error, and SGRT DIBH reproducibility error). In addition to the information on single couch direction displacement, the real-time AlignRT displacement vector magnitude (MAG) was calculated using Equation (1):

(1) $$MAG\;\left[cm\right]=\sqrt{X^{2}+Y^{2}+Z^{2}}$$

where *X*, *Y*, and *Z* represent shifts in lateral, longitudinal, and vertical directions, respectively. Couch shifts from each treatment session, applied after initial imaging with kV portal imaging (setup error) and shifts from control kV portal imaging during treatment fields (DIBH reproducibility) were also collected from patient treatment records. Differences in patient shifts based on gender were examined.

From the planning and control CT images, additional information on breath-hold variability was obtained by measuring distances between anatomical structures. During the treatment session, the patient took several deep breaths, with the number of breaths depending on the complexity of the plan and the volume of the irradiated area. Using the irradiation logs from the IBA control system (related to the beam control system), the average number and length of inspirations for each patient was determined. For this analysis, in-house Matlab 2016a (The MathWorks Inc., Natick, MA, USA) scripts were prepared.

#### Statistical Analysis

For patient position variability, the study group was analyzed for normality of distribution using the Shapiro–Wilk test [20], followed by the Mann–Whitney U test [25] with continuity correction, due to the small size of the study group and failure to achieve normal distribution of variables.

## 3. Results

### 3.1. Study I: FB vs. DIBH Treatment Plan Comparison

**Figure A1:**
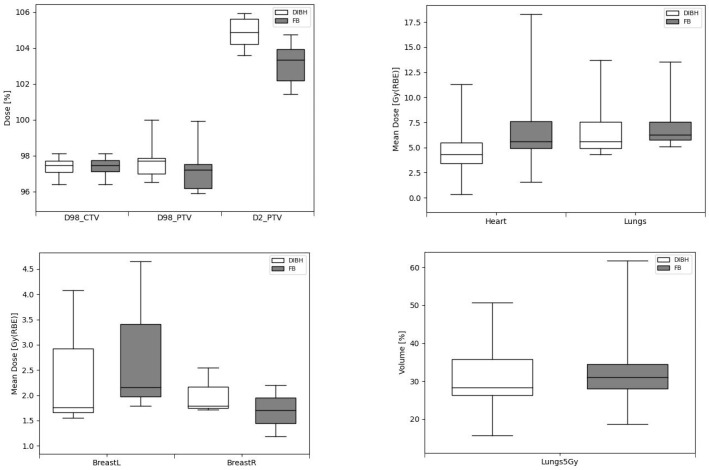
Dose distributions for PTV and selected OARs based on data from all patients. Box-and-whisker plots were employed to present the distributions (D98 CTV/PTV—dose to 98% of the CTV/PTV volume, D2 CTV/PTV- dose to 2% of the CTV/PTV volume, mean dose [Gy (RBE)]). Boxes expand from Q1 to Q3 quartile, median is visualized as the line in the box, whiskers depict minimum and maximum dose values.

Dose distributions from both plans were compared using selected DVH indices (Table A2, Appendix B). FB plans showed higher mean heart doses by an average of 2.5 Gy(RBE) (range: 0.2–7.02 Gy(RBE), median: 1.99 Gy(RBE)). Slight increases (<0.5 Gy(RBE)) were also noted in the lungs and left breast, while right breast doses were lower (mean: 0.3 Gy(RBE)) in the FB plans. Lung V5Gy increased by 3% on average (median: 2.43%). Dose values across patients are shown in Figure A1 (Appendix B).

Statistical analysis revealed a significant difference only for PTV D2% (*p* = 0.02). Other DVH indices showed no statistically significant differences (*t*-test *p* > 0.05; Wilcoxon test for mean lung dose: *p* = 0.16).

### 3.2. Study II: Verification of Inter-Fraction Breath-Hold Variability

Mean and maximum absolute differences in defined anatomical dimensions for six patients are presented in Table 2. All values are referenced to the planning CT. Across scans performed over a period of 27–36 days (from breathing training and initial low-dose scan to the final control CT after 5–10 treatment fractions), no consistent trend of increasing lung dimensions was observed.

#### 3.2.1. Breath-Hold Duration and Frequency per Radiation Field

In the analyzed patient group, the maximum breath-hold duration ranged from 49 to 106 s. Single-field beam delivery times ranged from 57 to 113 s, requiring between one and seven breath holds per field.

#### 3.2.2. Patient Position Variability

**Figure A2:**
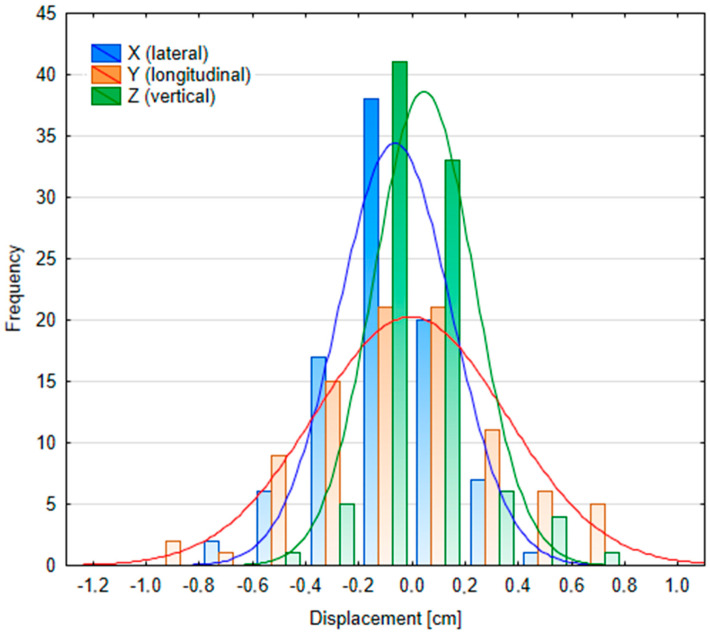
kV portal imaging SETUP errors (translations).

**Figure A3:**
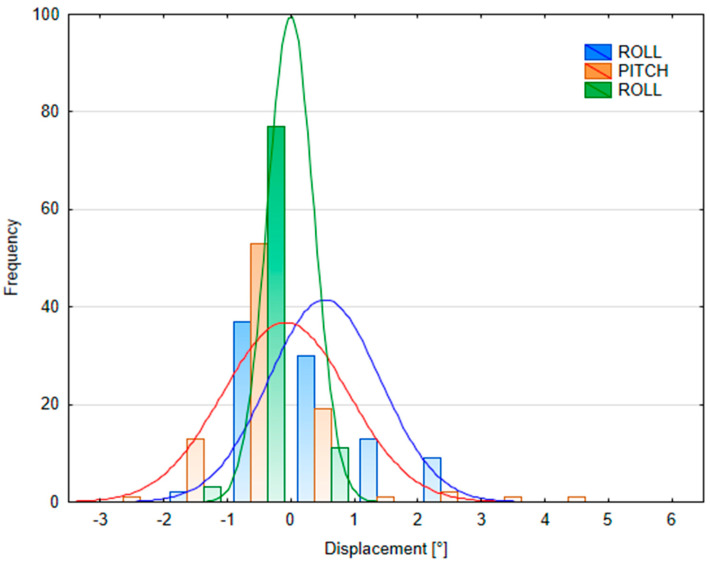
kV portal imaging SETUP errors (rotations).

**Figure A4:**
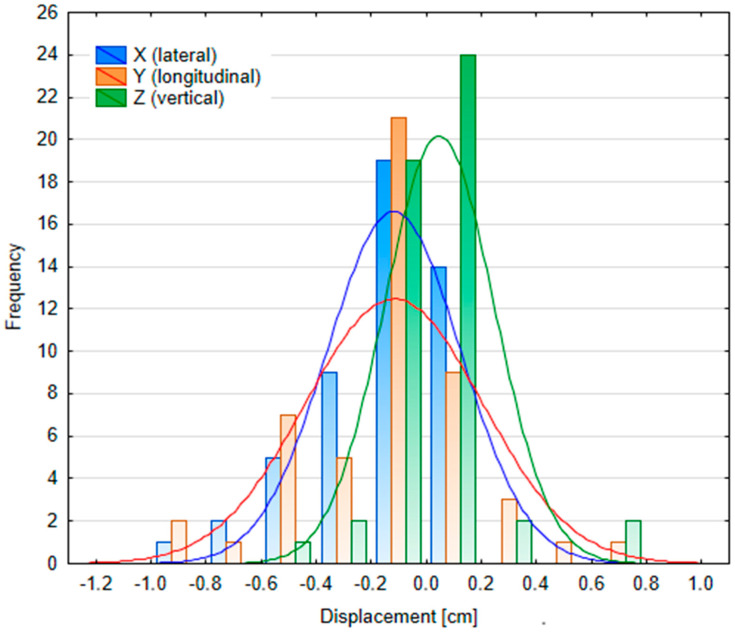
kV portal imaging DIBH reproducibility (translations).

**Figure A5:**
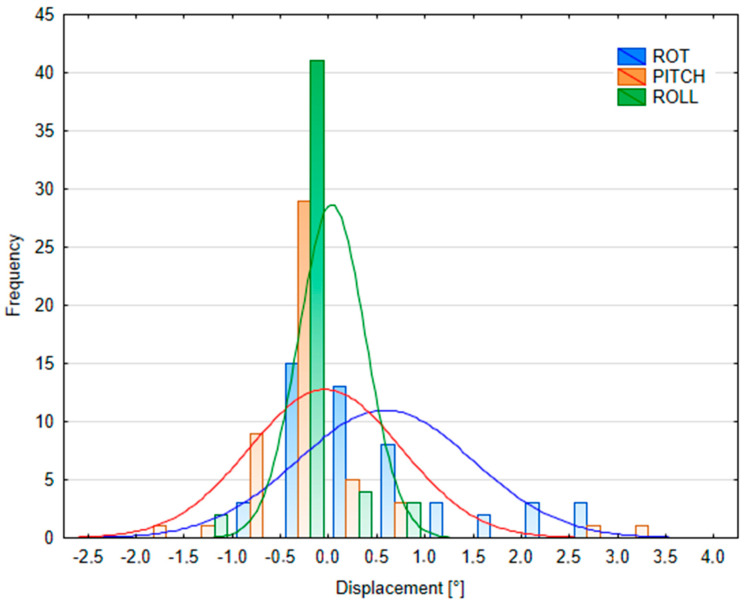
kV portal imaging DIBH reproducibility (rotations).

Data from optical cameras and kV X-ray systems with flat panel detectors were obtained separately for the same group of six patients across multiple positioning sessions. The analysis of SGRT data revealed statistically significant differences between male and female groups across several parameters. The results are presented in Table 3 (SGRT) and Table 4 (kV portal imaging). Histograms illustrating the translational and rotational shifts for all patients can be found in Figure A2, Figure A3, Figure A4 and Figure A5 in Appendix C.

During the initial patient setup using the FB body contour imported from the TPS into the AlignRT system, statistically significant differences were observed in vertical shifts (Z) and rotational corrections (pitch and roll) between male and female patients (*p* < 0.05). Male patients demonstrated lower median displacement values in the vertical axis (Z) and pitch rotation, whereas female patients showed lower median values for roll correction (see SGRT FB setup error in Table 3).

Statistically significant differences were also found during DIBH alignment in the lateral and vertical directions, as well as in rotational parameters, for both groups (*p* < 0.05). Male patients exhibited lower median values than female patients across all assessed parameters (see SGRT DIBH setup error in Table 3).

The reproducibility of the DIBH position using SGRT (based on the reference skin surface image after position correction) also showed statistically significant differences between male and female groups in the vertical (Z) direction and overall rotation (ROT), with female patients presenting lower median values. In contrast, male patients demonstrated lower median values in the lateral (X) and longitudinal (Y) directions, as well as in pitch and roll rotations (see SGRT DIBH reproducibility error in Table 3).

Positioning based on kV portal imaging using digitally reconstructed radiographs (DRRs) after patient alignment with AlignRT revealed statistically significant differences in setup errors between male and female patients. Male patients had lower median values for lateral (X) translation and overall rotation, while female patients showed lower median values for pitch rotation (see kV portal imaging setup error in Table 4).

In the control kV portal imaging data, statistically significant differences (*p* < 0.05) between male and female groups were observed in the lateral (X) direction and ROT, with male patients again presenting lower median values (see kV portal imaging DIBH reproducibility in Table 4).

However, when analyzing the magnitude of displacement across all parameters (from both SGRT and kV data), no statistically significant differences were found between male and female groups (*p* > 0.05). A summary of the displacement ranges for all patients is presented in Table 5. In the lateral and vertical directions, over 90% of cases exhibited a magnitude vector displacement within 5 mm, while for the longitudinal direction, more than 80% of cases were within this range. Differences in patient displacements, particularly in median shifts and rotations, may be attributed to variations in patient-specific characteristics such as height, weight, and body mass index (BMI) (see Table A3 in the Appendix D). The study group was analyzed with respect to these characteristics, revealing statistically significant differences between male and female patients in terms of age, height, and weight (*p* < 0.05).

These statistically significant differences highlight the heterogeneity of the study population. Therefore, the potential influence of patient characteristics on positioning accuracy should be investigated further in a larger cohort.

## 4. Discussion

Modern imaging techniques, such as optical systems for breath-hold monitoring, enable the treatment of mediastinal lymphoma patients with scanned proton beams. This study presents the first clinical experience with DIBH and proton beam therapy for mediastinal lymphoma. The process included DIBH-specific CT imaging, a patient training protocol, treatment planning, and retrospective analysis of both dose distributions and patient positioning during treatment. In addition to dosimetric outcomes, this study uniquely reports patient positioning results within a single group, which to the authors’ knowledge has not been previously presented. Furthermore, very initial potential differences between male and female subgroups were examined.

The methods and protocols implemented at CCB are consistent with the PTCOG Lymphoma Subcommittee recommendations [26]. As recommended, two separate CT scans were acquired for DIBH, including one with a low-dose protocol, as well as an FB scan. A beam model with increased layer spacing was also employed to reduce the number of monoenergetic layers, thereby shortening irradiation time—an important consideration for DIBH treatments. An optical imaging system was employed to monitor the patient surface, enabling safe and precise DIBH irradiation.

Previously published studies [26,27], along with the present findings, suggest that combining DIBH with proton therapy may provide advantages in sparing organs at risk (OARs), particularly the heart, compared with free-breathing (FB) irradiation. In the comparison between FB and DIBH plans, the mean average dose to the heart was found to be more than 2.5 Gy(RBE) higher in FB plans. No statistically significant differences were observed for other critical structures such as the lungs and breasts; however, the small sample size (*n* = 6) limits statistical power and hinders the ability to draw definitive conclusions. Nonetheless, FB plans required larger CTV and PTV margins, resulting in greater overall irradiated volumes compared with DIBH. Other studies support the dosimetric advantages of DIBH in proton beam therapy (PBS). For example, in [17], one of the earliest reports combining PBS and DIBH used patient-specific risk modelling and showed that DIBH significantly reduced the dose to cardiovascular structures by 2.3 Gy and to the lungs by 1.2 Gy (though the latter lacked statistical significance). DIBH was also associated with a lower predicted loss of life expectancy.

A similar analysis [28] compared photon and proton treatment plans based on FB and DIBH CT scans. That study reported reductions in dose to the heart and lungs by 38% and 13%, respectively, in DIBH proton plans compared with FB proton plans, with significant differences only in the lungs. There was no significant reduction in breast dose. Ultimately, among all tested modalities, DIBH with protons offered the most favorable dosimetric outcomes.

Another study [29] compared clinically prepared DIBH and FB proton plans using both pencil beam and Monte Carlo algorithms. DIBH was shown to reduce doses to the lungs, heart, and breasts by 2–5 Gy(RBE) compared to DIBH photon therapy. Compared to FB proton plans, DIBH reduced doses by up to 1.3 Gy(RBE). Importantly, assuming a variable RBE had limited impact on dose enhancement to cardiac structures.

In [30], a cohort of 13 patients was analyzed in terms of patient positioning using the SGRT system (AlignRT) and tumor position via kV cone-beam CT (CBCT). Median surface reproducibility with SGRT was reported at 0.9 mm (vertical), 1.3 mm (longitudinal), and 1.0 mm (lateral), with rotational deviations between 0° and 3.3°. These results align well with the present findings. In that study, rotational errors were generally lower, with median values under 1° and a maximum of 1.5°. The largest displacements were noted in the longitudinal (Y) axis, probably due to respiratory motion or table pitch variations.

The gender-based analysis showed statistically significant differences in patient displacements (based on SGRT and kV imaging), with females generally demonstrating higher values. Despite the fact the study’s cohort consisted of 6 patients, the positioning processes and their dependent data revealed statistically significant differences, according to non-parametric statistical tests. These differences may be attributable to patient characteristics such as height and weight. However, no significant difference in BMI was observed between groups, and prior research [31] has indicated that BMI, height, and weight do not significantly impact setup accuracy in head and neck or lung cancer patients. Thus, it remains unclear whether the higher displacement values in female patients reflect physiological differences or are coincidental in this analyzed cohort.

As highlighted previously [7], the use of PBS with motion management in mediastinal lymphoma is complex and requires meticulous preparation. As with the implemented protocol, the standard approach includes three CT scans for planning and a mid-treatment control scan. Treatment plans often use two to four fields (including RS when necessary), and extended layer spacing is employed to mitigate motion effects while reducing treatment time. Repainting was discontinued because it significantly prolonged treatment times, posing challenges for patients undergoing DIBH. Despite anatomical changes or breath-hold amplitude variations during treatment, the analysis of CT-based distances (Table 2) showed average changes below 8 mm (except for patient #4), remaining within the CTV-to-PTV margin. Due to the use of a low-dose protocol for control CTs, dose recalculation was not advised.

Clinical workflow for DIBH implemented at CCB is consistent with international standards and closely follows the approach recently described by Hörberger et al. [32]. That study showed that robust plans (D98% ≥ 95% for CTV) could be achieved with at least 10 of 12 perturbations using DIBH with PBS and surface imaging. Similarly, all clinical plans met the robustness criteria (D90% ≥ 95% for CTV) except for patient #2. Notably, the present findings confirm high reproducibility (median displacement < 2 mm via SGRT), which was previously identified as a concern but not quantitatively demonstrated [32].

Another important consideration in mediastinal lymphoma treatment planning is the relative biological effectiveness (RBE) of protons. While guidelines often assume a fixed RBE value, in practice, RBE may increase near the distal edge of the beam, especially near critical structures like the heart, lungs, and esophagus [33]. The actual high-dose region may therefore extend beyond what is predicted by standard treatment planning systems [34]. Monte Carlo simulations—especially those accelerated via GPU computing—offer a promising approach for better evaluating these effects and are currently being explored at CCB and by others [35]. Due to the high heterogeneity in the mediastinal region, incorporating Monte Carlo-based methods into proton planning is strongly recommended [29].

## 5. Conclusions

This study presents a combined clinical implementation of the DIBH method and its dosimetric and positioning outcomes in mediastinal lymphoma patients treated with PBS. The use of SGRT for patient positioning supports the clinical feasibility and accuracy of DIBH treatments, enabling precise, conformal, and OAR-sparing proton therapy. Although the gender-based analysis revealed some statistically significant differences, further investigation in a larger patient cohort is needed to determine and ensure the impact of gender on positioning reproducibility and treatment setup, due to the limited strength of statistical analysis based on this limited cohort size. It should therefore be emphasized that the results presented in this paper should be interpreted as preliminary evidence rather than broadly generalized conclusions.

## Figures and Tables

**Table 1:** Demographic and treatment characteristics of analyzed patients.

Patient ID	#1	#2	#3	#4	#5	#6
Age	27	38	40	26	25	35
Gender	male	male	male	female	female	female
Diagnosis/ Tumor subtype	HL, NS	HL, NS	PMBCL	HL, NS	HL, NS	HL, NS
Stage	1 A	2 A	2 BX	2 BX	2 A	4
Chemotherapy	4 × ××ABVD	× x ABVD	× x R-CHOP-14 × x ESHAP CAR-T	× x ABVD	× x ABVD	× x ABVD
PTV volume DIBH/FB [cm^3^]	545.65/601.93	516.04/680.54	216.67/306.0	1034.38/1301.35	772.56/871.39	377.03/513.92
Fraction dose/no of fractions	2.0 Gy(RBE)/15	2.0 Gy(RBE)/15	2.0 Gy(RBE)/ 25	2.0 Gy(RBE)/15	2.0 Gy(RBE)/15	2.0 Gy(RBE)/15
Beam arrangement	0° RS/0°/330° RS/30° RS	0° RS/0° RS/330°/30°	0° RS/0° RS/20° RS/340° RS	0° RS/0° RS/340° RS/340° RS/13° RS	0° RS/0° RS/20° RS/340° RS	0° RS/0° RS/343° RS/17° RS

Abbreviations: HL: Hodgkin lymphoma, NS: nodular sclerosis, BX: biopsy, ESHAP: etoposide + methylprednisolone + cytarabine + cisplatin, CAR-T: chimeric antigen receptor T-cell therapy, RS: range shifter.

**Table 2:** Mean and maximum differences in measured distances relative to the planning CT.

Distance	#1		#2		#3		#4		#5		#6	
	Mean	Max	Mean	Max	Mean	Max	Mean	Max	Mean	Max	Mean	Max
∆A [cm]	0.27	0.56	0.29	0.45	0.41	0.68	0.07	0.28	0.01	0.02	0.06	0.34
∆B [cm]	0.70	0.90	0.37	0.77	0.45	1.54	0.88	1.58	0.23	0.57	0.32	1.51
∆C [cm]	0.29	1.31	0.38	0.55	0.03	0.56	0.09	0.24	0.10	0.43	0.78	1.87

**Table 3:** Median shifts obtained using the SGRT system grouped by gender. Values in brackets represent quartiles Q1 and Q3.

Table Shift Coordinates	Median SGRT FB Setup Error			Median SGRT DIBH Setup Error			Median SGRT DIBH Reproducibility Error		
	Male (*n* = 58)	Female (*n* = 46)	*p*-Value	Male (*n* = 49)	Female (*n* = 40)	*p*-Value	Male (*n* = 62)	Female (*n* = 69)	*p*-Value
X (lateral) [cm]	0.02 (−0.1; 0.04)	0 (−0.06; 0.11)	*p* = 0.06	−0.08 (−0.14; 0)	0.08 (−0.03; 0.23)	*p* < 0.05	0 (−0.05; 0.03)	0.03 (−0.01; 0.08)	*p* < 0.05
Y (longitudinal) [cm]	0.03 (−0,07; 0,13)	0.08 (−0.07; 0.16)	*p* = 0.27	−0.24 (−0.54; −0.02)	−0.25 (−0.43; −0.15)	*p* = 0.72	0.02 (−0.12; 0.13)	0.11 (0.01; 0.19)	*p* < 0.05
Z (vertical) [cm]	0.08 (0.01; 0.13)	0.12 (−0.06; 0.21)	*p* < 0.05	−0.1 (−0.2; 0.06)	−0.16 (−0.27; −0.09)	*p* < 0.05	0.07 (−0.02; 0.13)	0.02 (−0.01; 0.07)	*p* < 0.05
MAG (magnitude) [cm]	0.17 (0.14; 0.25)	0.24 (0.15; 0.29)	*p* = 0.54	0.41 (0.22; 0.68)	0.40 (0.23; 0.54)	*p* = 0.38	0.19 (0.13; 0.27)	0.17 (0.12; 0.23)	*p* = 0.26
ROT [°]	0 (−0.44; 0.37)	0.1 (−0.27; 0.53)	*p* = 0.24	0.23 (−0.02; 0.38)	−0.95 (−1.44; −0.12)	*p* < 0.05	0.22 (0.07; 0.35)	0.03 (−0.19; 0.29)	*p* < 0.05
PITCH [°]	0.05 (−0.75; 1.17)	−0.97 (−1.72; −0.23)	*p* < 0.05	0.47 (−0.08; 1.21)	0.06 (−1.14; 1.23)	*p* = 0.07	−0.07 (−0.35; 0.3)	−0.28 (−0.61; 0.02)	*p* < 0.05
ROLL [°]	0.18 (−0.26; 0.69)	−0.13 (−0.70; 0.52)	*p* < 0.05	−0.04 (−0.17; 0.17)	−0.21 (−0.77; 0.05)	*p* < 0.05	0.05 (−0.06; 0.16)	0.19 (0.05; 0.28)	*p* < 0.05

**Table 4:** Median shifts obtained using kV portal imaging (DRR matching) grouped by gender. Values in brackets represent quartiles Q1 and Q3.

Table Shift Coordinates	Median kV Portal Imaging Setup Error			Median kV Portal Imaging DIBH Reproducibility		
	Male (*n* = 45)	Female (*n* = 38)	*p*-Value	Male (*n* = 17)	Female (*n* = 33)	*p*-Value
X (lateral) [cm]	0 (−0.06; 0.10)	−0.14 (−0.30; 0)	*p* < 0.05	0 (−0.06; 0.03)	−0.09 (−0.34; 0)	*p* < 0.05
Y (longitudinal) [cm]	0.02 (−0.14; 0.24)	−0.03 (−0.34; 0.12)	*p* = 0.16	0 (−0.15; 0.06)	−0.07 (−0.45; 0)	*p* = 0.13
Z (vertical) [cm]	0 (−0.06; 0.11)	0.01 (−0.06; 0.12)	*p* = 0.74	0.05 (−0.1; 0.09)	0.02 (0; 0.12)	*p* = 0.39
MAG (magnitude) [cm]	0.34 (0.16; 0.43)	0.33 (0.17; 0.68)	*p* = 0.18	0 (0; 0.08)	0 (0; 0.26)	*p* = 0.06
ROT [°]	0 (−0.2; 0.4)	0.65 (0; 1.5)	*p* < 0.05	0 (−0.2; 0.3)	0.5 (0; 1.5)	*p* < 0.05
PITCH [°]	−0.31 (−0.93; 0)	−0.05 (−0.3; 0.5)	*p* < 0.05	−0.27 (−0.5; 0)	0 (−0.21; 0.07)	*p* = 0.11
ROLL [°]	0 (0; 0)	0 (0; 0)	*p* = 0.09	0 (0; 0)	0 (0; 0)	*p* = 0.43

**Table 5:** The percentage of positionings for different translational thresholds.

Tolerances Within	kV Portal Imaging Setup Error (Interfractional, *n* = 91)			kV Portal Imaging DIBH Reproducibility (Intrafractional, *n* = 48)		
	X	Y	Z	X	Y	Z
1 mm	45.1%	34.1%	50.5%	45.8%	41.7%	56.3%
2 mm	63.7%	46.2%	80.2%	64.6%	56.3%	85.4%
3 mm	82.4%	58.2%	87.9%	75.0%	66.7%	91.7%
4 mm	90.1%	74.7%	93.4%	83.3%	75.0%	93.8%
5 mm	96.7%	82.4%	97.8%	93.8%	85.4%	93.8%

## Data Availability

All data generated in this study are available from the corresponding author upon request.

## Abbreviations

The following abbreviations are used in this manuscript:

ABVD	adriamycin + bleomycin + vinblastine + dacarbazine
BEACOPP	bleomycin + etoposide + doxorubicin + cyclophosphamide + vincristine + procarbazine + prednisolone
BH	breath -hold
BX	biopsy
CAR-T	chimeric antigen receptor T-cell therapy
CCB	Cyclotron Centre Bronowice
CT	computed tomography
CTDIvol	computed tomography dose index volume
CTV	clinical target volume
DIBH	deep inspiration breath hold
DRR	digitally reconstructed radiograph
DVH	dose-volume histogram
ESHAP	etoposide + methylprednisolone + cytarabine + cisplatin
FB	free breathing
GTV	gross tumor volume
HL	Hodgkin lymphoma
IBA	ion beam application
ITV	internal tumor volume
kV	kilovoltage
NS	nodular sclerosis
OAR	organ at risk
PBS	pencil beam scanning
PMBCL	primary mediastinal B-Cell lymphoma
PT	proton therapy
PTV	planning target volume
RS	range shifter
SGRT	surface-guided radiotherapy
TPS	treatment planning system
UBTI	universal beam triggering interface
UTU	universal trigger unit

## Appendix A

CT parameters

**Table A1:** Scanning protocol parameters for full-dose and low-dose CT imaging.

Parameter	Value
Scanning technique	Axial
Scanning direction	Caudo-cranial
Tube voltage (kV)	120 (full-dose), 70 (low-dose)
Tube current-time product (mAs)	CareDose4D (qual.ref. 190 mAs)
Slice (increment)	2 mm(2 mm) acq. 64 × 0.6 mm
Feed	17 mm
Scan time	0.36 s (quick)
Cycle time	1.25 s
Delay	2 s
Field of view (FOV)	500 mm
Reconstruction kernel	B30s medium smooth

## Appendix B

Statistical analysis—dosimetric comparison DIBH vs. FB

**Table A2:** DVH indices, clinical goals and results for both planning methods—DIBH and FB.

DVH Parameter	Goal	#1			#2			#3			#4			#5			#6		
		DIBH	FB	delta	DIBH	FB	delta	DIBH	FB	delta	DIBH	FB	delta	DIBH	FB	delta	DIBH	FB	delta
D98% ITV/CTV [%]	>95%	97.28	97.28	0.00	97.02	97.05	0.03	97.60	97.60	0.00	97.75	97.79	0.04	98.10	98.10	0.00	96.40	96.40	0.00
D98% PTV [%]	>95%	100.00	99.94	−0.06	96.75	95.94	−0.81	97.66	97.50	−0.16	97.78	97.51	−0.27	97.88	96.91	−0.97	96.53	95.89	−0.64
D2% PTV [%]	<105%	104.21	101.42	−2.79	105.94	104.75	−1.19	104.24	102.88	−1.36	105.52	103.81	−1.71	103.60	103.97	0.37	105.63	101.96	−3.67
D̅Heart [Gy(RBE)]	<5 Gy *	3.74	6.15	2.41	3.33	4.90	1.57	0.34	1.59	1.25	11.29	18.31	7.02	4.86	5.06	0.20	5.70	8.10	2.40
D̅Lungs [Gy(RBE)]	<10 Gy **	5.23	5.97	0.74	4.86	5.69	0.83	4.34	5.11	0.77	13.68	13.54	−0.14	8.06	7.90	−0.16	5.97	6.61	0.64
V5GyLungs [%]	<55%	27.21	28.70	1.49	25.98	27.80	1.82	15.66	18.69	3.03	50.78	61.82	11.04	37.88	34.89	−2.99	29.40	33.50	4.10
D̅Breast(L) [Gy(RBE)]	<3 Gy	-	-	-	-	-	-	-	-	-	4.08	4.65	0.57	1.76	1.79	0.03	1.55	2.16	0.60
D̅Breast(R) [Gy(RBE)]	<3 Gy	-	-	-	-	-	-	-	-	-	2.55	2.20	−0.35	1.79	1.19	−0.61	1.71	1.71	0

* Mean dose <8 Gy (recommended), mean dose <15 Gy (acceptable). ** Mean dose <13.5 Gy (recommended).

## Appendix C

Statistical analysis—patient position variability

## Appendix D

Patient characteristics

**Table A3:** Patient characteristics grouped by gender.

Characteristic	Median: Male (*n* = 3)	Median: Female (*n* = 3)	Z	*p*-Value
Age [years]	40 [38; 40]	26 [25; 35]	Z = 13.72	*p* < 0.05
Height [m]	1.69 [1.69; 1.73]	1.65 [1.62; 1.70]	Z = 10.60	*p* < 0.05
Weight [kg]	65 [65; 71.5]	63 [59; 93.9]	Z = 7.24	*p* < 0.05
BMI	22.76 [22.76; 23.36]	23.14 [22.48; 32.47]	Z = −0.43	*p* = 0.66
